# Indian psychiatry, research and Asian countries

**DOI:** 10.4103/0019-5545.69214

**Published:** 2010-01

**Authors:** J. K. Trivedi, Pawan Kumar Gupta, Rahul Saha

**Affiliations:** Department of Psychiatry, C.S.M. Medical University, (erstwhile K.G. Medical University), Lucknow - 226 003, Uttar Pradesh, India

**Keywords:** Psychiatric research, Asian countries, global psychiatry

## Abstract

Asia has some of the largest conglomerations of human populations and also the fastest growing economies of the world. About 23% of the world’s population lives in the South Asian region, and one-fifth of psychiatrically ill patients in the world live in this part of the world. Despite vast cultural, religious, geographical, and political diversities, the factors influencing mental health remain the same throughout this wide region, as highlighted at the recently concluded Asian summit, where the slogan, ‘One vision, one identity, one community,’ was launched. Thus, the need to strengthen regional cooperation in the field of mental health has always been felt. This article highlights facts about influence of Indian Psychiatry research as well as of some Asian countries in the world psychiatry and vice versa.

## INTRODUCTION

Asia has some of the largest conglomerations of human populations and also the fastest growing economies of the world. About 23% of the world’s population lives in the South Asian region, and one-fifth of psychiatrically ill patients in the world live in this part of the world. The South Asian Association for Regional Co-operation (SAARC) was formed on December 8, 1985, in Dhaka, Bangladesh. The seven member states are Bangladesh, Bhutan, India, Maldives, Nepal, Pakistan, and Sri Lanka. Afghanistan has become a new member since 2005.

Despite vast cultural, religious, geographical, and political diversities, the factors influencing mental health remain the same throughout this wide region, as highlighted at the recently concluded Asian summit, where the slogan, ‘One vision, one identity, one community,’ was launched. Thus, the need to strengthen regional cooperation in the field of mental health has always been felt.

## HISTORY OF INDIAN PSYCHIATRY

The medical compendia of ancient India are replete with references to mental health, covering a spectrum of diagnoses, classification, and treatment methods. A description of insanity — *unmada* (oonma-tha) — dating back to 1500 BC, exists in the *Atharva Veda*, the most ancient authentic Indian medical scripture. Descriptions of conditions similar to schizophrenia and bipolar disorder appear in the Vedic texts; these texts differentiate doctors practicing magical medicine from scientific physicians and surgeons, who lived and practiced in cottages surrounded by medicinal plants. An ancient textbook of Ayurvedic medicine, *Therapeutics and Surgical Practice* by Charaka and Susrutha, has a vivid description of schizophrenia. It states clearly that only an expert in the field of mental health should treat people with this illness. Other traditional medical systems, such as Siddha, which recognizes various types of mental disorders, flourished in southern India. The great epics such as the *Ramayana* and the *Mahabharata* made several references to the disordered states of mind and means of coping with them. The Bhagavad Gita is a classical example of crisis intervention psychotherapy.[1]

Till about seventeenth century all abnormal behavior was believed to be act of the ‘devil,’ that is, ‘Against God’. The ‘Mentally ill’ were considered evil and described as witches. Gradually over passing time, mental illness was considered as a ‘deviant behavior’ and mentally ill were considered socially unacceptable and put in jails along with other criminals. In the modern era, there was a shift from ‘evil’ to ‘ill’. Mentally ill were called ‘mad’ or ‘insane’ and were placed in special places called ‘asylums’. However, gradually these asylums became the place for human exploitation. Phillipe Pinel was the first Psychiatrist to free these mentally ill from the asylum. Clifford Beers work, ‘The mind that found it self,’ brought to light the treatment meted out to these people in asylums, resulting in a strong reaction to the plights of the mentally ill. This uproar resulted in the starting of the ‘mental-hygiene’ movement.

In the twentieth century, the work of Freud and ‘B. F. Skinner and J. B. Watson’ gave a scientific combination of biological and social theories to explain the etiology of mental illness.

## PSYCHIATRIC EPIDEMIOLOGY IN INDIA WHICH INFLUENCED WORLD

Psychiatric epidemiology has gone through various stages of growth over the past five decades in India, starting from the first psychiatric epidemiological study by Dube,[2] in 1961, at Agra, to the development of tools like the Present Status Examination (PSE)[3] and the Indian Psychiatric Survey Schedule (IPSS).[4] A major advance in psychiatric epidemiology is the development of reliable and valid diagnostic interviews.

The International Pilot Study of Schizophrenia[5] and the Determinants of the Outcome of Severe Mental Disorders (DOSMED) study,[6] have provided convincing evidence for a better outcome in India (along with other less industrialized countries) than in the West. This finding of a good outcome of treatment also emerged in the Chandigarh studies,[7,8] which showed that regardless of the diagnostic definition, the outcome in Indian patients was favorable. One spin-off of the IPSS, in India, was the multi-site Study of Factors Affecting the Course and Outcome of Schizophrenia (SOFACOS), sponsored by the Indian Council of Medical Research. This was a five-year follow-up of 386 patients in three centers, aimed at identifying the social and clinical factors affecting the course and outcome. The two-year follow-up revealed that among the 423 patients who were followed up, 64% were in remission and only 11% continued to be ill.[9] Data from the Madras Center also revealed a better outcome for women at the end of five years of follow-up.[10]

The Madras cohort was followed up for another 10 years and it was found that it had a better outcome in marital and work functioning than has been reported for similar populations in the West.[11,12] Another interesting aspect of the Madras study has been the quality of social support enjoyed by the cohort. All the patients continued to live at home and were cared for by either their spouse or their parents. This can be generalized to other parts of the country, where the concept of patients living alone because of their illness still meets with considerable social disapproval. However, the increase in nuclear families and the reduction in the number of joint families, increasing urbanization, and the changing role of women may all, unfortunately, alter this picture in the years to come.

In India, relapse is difficult to ascertain. Rehospitalization is one of the chief criteria of relapses in the West, but is not so here.[13] This is due to a combination of factors such as lack of beds and personnel, the costs involved, and the reluctance of the family in some cases to have the patient repeatedly admitted. Costs also determine the type of medication, and many patients are still given conventional antipsychotic drugs.

The role of the family in the Indian scene is all-pervasive, influencing as it does the decision to seek help (when, where, and how), the nature of help (medical or nonmedical), the need to continue treatment, and other issues such as employment and marriage. A report by Srinivasan and Thara[14] describes the role of the family in dealing with acutely ill patients with schizophrenia, who are unwilling to take medication.

The recognition of the importance of families has led to the creation of facilities for families to stay with patients in some centers, such as the National Institute of Mental Health and Neurosciences in Bangalore, and the Christian Medical College in Vellore. This facilitates the extensive participation of families in therapeutic programs. The burden on families of people with mental illness is emotional, physical, financial, and medical. Two instruments have been developed specifically to study this burden.[15,16] Coping by families has also received some attention, with particular reference to religious coping methods.[17]

## ROLE OF INDIAN PSYCHIATRY IN SOUTH ASIA

South Asia has been lacking in the field of mental health research mainly due to lack of adequate financial support and infrastructure, and poor collaboration among various health agencies in the region, due to political barriers among the countries.

The whole of South Asia faces the problem of ‘inequalities in health’. The majority of people live in rural areas or urban slums, with no access to care. New delivery systems are needed to target this large group. Pathways to care should be determined and traditional sources (e.g., magico religious healers) should be explored.

Some potential areas for research collaboration in South Asia have been identified: psychiatric rehabilitation, treatment of major depression, culturally acceptable psychotherapy, delivery of mental health services, epidemiological studies, burden of care, course and outcome of mental disorders, acute psychoses, classificatory systems, psycho education, and transcultural psychopharmacology.

Individual countries like India have made some significant contributions in psychiatric research. The work of various agencies has been commendable, and the Indian Council of Medical Research, the World Health Organization, the Department of Science and Technology of the Indian Government, the Indian Council of Scientific and Industrial Research, and the United Nations Children’s Fund have been noteworthy among them. Various psychiatric associations have been formed, such as, the Asian Federation for Psychiatry and Mental Health (AFPMH), in 1981, (Indonesia, Malaysia, Philippines, Thailand, Singapore, now also Brunei, Laos, Cambodia, Myanmar, Vietnam), the South Asian Forum for Psychiatry and Mental Health (SAFPMH), in 2002, (India, Pakistan, Sri Lanka, Bangladesh, Nepal, Bhutan), and the SAARC Federation of Psychiatry (SFP), in 2004. The global pharmaceutical companies are also targeting India, by involving several Indian institutions in various international multicentric studies. Also noteworthy is the contribution of the Indian Psychiatric Society (IPS), as it is the largest ensemble of trained psychiatrists in South Asia and has a major role in coordinating research in the region. The Society has promoted the participation of the whole region in its annual conferences, in order to promote better intra-regional cooperation in research.

## ROLE OF THE INDIAN PSYCHIATRIC SOCIETY

Our society sprouted from the Indian Association for mental hygiene founded in 1929, by Berkeley Hill. In 1935, the Indian division of the Royal Medico-Psychological Association was formed due to the efforts of Dr. Banarasi Das. Thanks to the efforts of Dr. Nagendra Nath De, Major R. B. Davis, and Brigadier T. A. Munro, the association gained its new name, the Indian Psychiatric Society (IPS) on 7 January, 1947. The rules and regulations were framed by the eminent Psychiatrists of that period (Dhunjibhoy, Rosie, Kenton, Llyodo, Masani, Shah, Johnson, Govindaswamy, and Kak). The first annual meeting held on 2 January, 1948, at Patna was presided by N.N De.[18,19] The society has enlarged into a group of 3000 fellows (right of franchise) and many ordinary members. The activities of the IPS are also delegated to the state branches, coming under five zones.

The achievements include academic updates as part of professional development, publications like IDEAS[20] and Clinical Practice Guidelines, and the Indian Journal of Psychiatry. Some efforts have been made for mental health literacy and community service strategies. Although psychiatrists are involved in the NMHP (National Mental Health Program) and DMHP (District Mental Health Program), the society’s involvement as a stakeholder is still not appreciated.

The Indian Psychiatric Society is a member of the WPA (World Psychiatric Association) and the SAARC psychiatric federation. The SAARC psychiatric federation comes under the Asian Federation of Psychiatric Association (AFPA). IPS has links with the Indo-American Psychiatric Association, Indo-Australian Psychiatric Association, British Indian Psychiatric Association, and the Indo-Canadian Psychiatric Association. Another organization involved in the training and service delivery representing 18 countries is the South Asian Forum International (SAFI).

The collaboration and the goodwill of these bodies should be encouraged for the benefit of Indian Psychiatry. The Royal College of Psychiatrists can assist in teaching and training, in collaboration with IPS.

## FINAL WORDS OF WISDOM

India is a multicultural, multi-ethnic, pluralistic society with enormous socioeconomic disparities. This variety on the one hand is exciting, stimulating much research in the field of behavior and mental health, and on the other hand, it is a daunting task to provide affordable and effective mental healthcare, especially to the remote rural corners of the country. The low budget accorded to health and the unenviably low priority of mental health does not make this task any easier.

But yet, various studies in India, such as IPSS and DOSMED, have given the world a different outlook toward the role of family in psychiatric illnesses and helped in bringing out various forms of therapies involving family members, which significantly affect the outcome of patients with chronic mental illness. India has played a pivotal role in leading from the front in the region of south Asia in the field of research and patient care, and guiding other countries in psychiatry care.

This is clearly the most exciting time in history for the field of psychiatry. The expansion of our knowledge base, as the result of advances in clinical description, neuroscience, and other areas related to etiology and treatment have led to important developments in our approaches to the diagnosis and treatment of individuals with psychiatric illnesses. For example, there has been an increasing recognition of the impact of cultural influences and spiritual beliefs as important factors influencing presentation of illness and symptomatology.

The increasing number of mental health professionals, the availability of newer drugs, access to information, and the presence of a few centers of excellence augur a bright future for the mental health scene in India, which will be another step in developing the field of psychiatry. This will provide the vitally needed qualitative thrust to psychiatric teaching and research.
